# XR-integrated brain-computer interfaces for augmentative and alternative communication: a systematic review

**DOI:** 10.3389/fnhum.2026.1842938

**Published:** 2026-07-15

**Authors:** Peiran Zhang, Petra Karlsson, Darryl Chiu, Ran Zou, Cheng Zhang, Alistair McEwan, Bomin Sun, Haifeng Zhao

**Affiliations:** 1 Department of Neurosurgery, Ruijin Hospital, Shanghai Jiao Tong University School of Medicine, Shanghai, China; 2 Children’s Hospital at Westmead Clinical School, The University of Sydney, Sydney, NSW, Australia; 3 Research Institute, Cerebral Palsy Alliance, Sydney, NSW, Australia; 4 School of Biomedical Engineering, Faculty of Engineering and Information Technology, The University of Sydney, Sydney, NSW, Australia; 5 Institute of Medical Artificial Intelligence, Shanghai Jiao Tong University School of Medicine, Shanghai, China; 6 Clinical Neuroscience Center, Ruijin Hospital, Shanghai Jiao Tong University School of Medicine, Shanghai, China

**Keywords:** augmentative and alternative communication (AAC), brain–computer interface (BCI), cerebral palsy (CP), systematic review, virtual reality (VR)

## Abstract

**Introduction:**

Communication is a fundamental human right and should not be constrained by disability; advances in modern technology are reshaping how communication is conceived and delivered, creating new possibilities for people who rely on augmentative and alternative communication (AAC). This systematic review examined whether immersive virtual, augmented, and mixed reality (VR/AR/MR; hereafter referred to as XR) has been integrated with brain-computer interfaces (BCIs) to support AAC for people who rely on it, and what evidence exists regarding usefulness and usability.

**Methods:**

Searches were conducted in five databases, with the initial search completed on 30 June 2021 and the updated search completed on 18 November 2025.

**Results:**

Two eligible studies were included. The evidence base was limited and methodologically heterogeneous, spanning distinct integration pathways: (i) an immersive-display implementation of a classic P300 speller suggesting communication performance comparable to conventional displays; and (ii) a wearable mixed-reality AAC concept contrasting non-BCI access (eye-gaze) with BCI-based access, with non-BCI access appearing more practically usable while BCI access remained challenging. Overall, the included studies indicate that immersive XR platforms can technically accommodate BCI-mediated interaction for communication-related purposes.

**Discussion:**

Current evidence remains sparse and heterogeneous, with usability and usefulness varying by application target and evaluation endpoint. This review therefore synthesises the available feasibility and usability/usefulness evidence and offers an open discussion of design and evaluation priorities for advancing BCI-enabled immersive AAC toward practical use in people who rely on it.

**Systematic review registration:**

https://www.crd.york.ac.uk/prospero/display_record.php?ID=CRD42021273338, identifier: CRD42021273338.

## 1 Introduction

Conveying information—communication—is a primitive freedom and basic human right (McEwin and Santow, 2018). The convention of exchanging information is redefined by modern technology. Modern technology has made communication more convenient and efficient than before (Alhadlaq, 2016). Moreover, it also provides inclusiveness to people who have complex communication needs (McLeod, 2018). Augmentative and Alternative Communication (AAC) is an umbrella term referring to a range of tools, strategies, and technologies designed to support, supplement, or replace spoken and written communication for individuals who have difficulty using conventional communication methods.

The original motivation of this study was to explore the currently available AAC devices for people with cerebral palsy (CP). CP is a neural disorder that is usually caused by brain damage in an early stage of a person’s life. The current overall birth prevalence of cerebral palsy was 1.6 per 1,000 live births in high-income countries (McIntyre et al., 2022). It may cause various levels of motor impairments associated with difficulties and/or disorders in sensation, cognition, communication, perception and behaviour (Rosenbaum et al., 2007). The communication capability of half the population of people with CP are affected and about 30% are non-verbal (Nordberg et al., 2013). AAC has been provisionally accepted to be effective and efficient communication assistance and rehabilitation method for people with CP and in communication needs (Smith and Hustad, 2015; Avagyan et al., 2021; Smith and Connolly, 2008). Moreover, it was found that an earlier AAC intervention is more beneficial (Romski et al., 2015).

AAC is not an exclusive technology in interventions for CP. It is also broadly used for therapies or assistive instruments to Autism Spectrum Disorder (ASD) (Lima Antao et al., 2018), Amyotrophic Lateral Sclerosis (ALS) (Hanson et al., 2011) and other types of speech impairments (Teachman and Gibson, 2014). ASD is also a neurodevelopmental condition typically emerging in early childhood. ALS, on the other hand, is a progressive neurological disorder that will affect the patient’s speech at a certain stage of the development of this disease (Strong et al., 1996). AAC is used across a range of conditions associated with communication disability, including CP, ALS, and subsets of autistic people with limited functional speech. Since these AAC applications are transferable, variant impairments that may produce complex communication needs were included in this review’s scope.

One of the common components of AAC devices is an interactive interface that provides pictures, vocabulary or sentence candidates for people to select (Murray and Goldbart, 2009). The conventional display of this interface is usually based on a low-tech physical board or high-tech screen monitor (Murray and Goldbart, 2009). Although the current design could provide some degrees of accessibility for people who rely on AAC to regain their communication ability, the privacy, portability and adaptability of such device was warranted by the users (Cooper et al., 2009; McNaughton and Bryen, 2007). Accordingly, Alm et al. (1998) explored the use of three-dimensional (3D) virtual scenes as a potential interface for complex computer-based communication systems for non-speaking individuals, suggesting an early connection between immersive interaction and AAC-related access. The fundamental unit of VR is the interactive virtual object that users can view and manipulate as it is a real object in the life (Sherman and Craig, 2003). Consequently, VR can provide an immersive interaction between humans and computers. In addition to virtual interactive object, augmented reality (AR) also utilise the real world as background (Carmigniani and Furht, 2011). Users may have a more intuitive experience in AR because virtual objects or information can be situated within their familiar physical environment, rather than being presented on a separate communication board or screen, which may support more contextually embedded and engaging interaction (Krichenbauer et al., 2018). Mixed reality (MR) is often confused with AR, although the two concepts can be distinguished conceptually (Lungu et al., 2021). According to Lungu et al. (2021), AR mainly overlays digital information onto the real world, whereas MR embeds virtual objects within it. For example, Google Glass overlays information onto the user’s view of real-world objects, whereas Microsoft HoloLens uses holographic technology to present virtual objects within the real-world environment (Kalantari and Rauschnabel, 2018). However, AR and MR are sometimes used interchangeably in the literature to describe technologies that combine virtual and real-world elements (Perkins et al., 2017; Zhao et al., 2021). To simplify the terminology, this paper uses XR as an umbrella term for VR, AR, and MR, unless a specific type needs to be clarified.

Using XR as an intervention has shown potential benefits for communication development among people with special needs (Cobb, 2007). XR-based interventions may offer bidirectional benefits: they can support users by reducing stress or anxiety in therapeutic contexts (Fernandez-Aranda et al., 2012), while also helping researchers and clinicians better understand users’ behaviours and responses in controlled simulated environments (Bekele et al., 2013). In particular, VR interventions have been used to facilitate communication skills (Herrero and Lorenzo, 2020; Ghanouni et al., 2021) and social interaction (Welch et al., 2009; Zhao et al., 2018) among people with ASD. Several studies have developed VR-based social scenarios to support social communication, interaction, or behavioural training for individuals with ASD (Lahiri et al., 2011a; Lahiri et al., 2011b; Lahiri et al., 2011c; Kuriakose et al., 2012; Lorenzo et al., 2013; Lahiri et al., 2015; Babu et al., 2018; Raj and Lahiri, 2017; Krishnappa Babu and Lahiri, 2024). These studies suggest that XR can provide controlled, repeatable, and context-rich environments for communication-related interaction, which provides a rationale for considering XR as a potential interface layer for AAC.

Building on this rationale, several XR-based systems have been explored to support communication-related interaction, particularly for people with ASD. In 2014, Wang et al. (2014) designed an AR-based augmentative communication device that drew a prompt over the face of the person whom the user is talking to. This method could boost the communication engagement for people with ASD (Wang et al., 2014). Similarly, in 2018, the Vahabzadeh et al. (2018) also arranged a pilot study to affirm that their augmentative communication device could mitigate attention problems for young adults with ASD. In the other paper for the same study, they also mentioned that the safety and lack of negative effects were the merits of using wearable AR-based device (Sahin et al., 2018). Compared with Wang et al. (2014), Xu et al. (2015) nominated a more complicated design, LittleHelper, which intended to amend the users’ behaviour such as raising/lowering their voices and re-orienting their spot towards the talking target. This design was believed to be helpful for the job interviews for people with ASD. The other system, VrSocial, from Boyd et al. (2018) was analogous to Little Helper. The difference was that the VrSocial was on VR instead of AR and it was more about training instead of real-time communication enhancement. With respect to the social cues understanding, Sun et al. (2019) suggested an emoji rendering solution. Their AR-based glasses could detect a head-worn image marker and rendered an emotion-presenting 3D emoji, which was considered to be beneficial to the facial expression understanding of people with ASD (Sun et al., 2019).

Notwithstanding, these studies were all for augmentative communication but lacked alternative communication. For people who rely on AAC, especially those with severe motor impairments and minimal functional speech, the missing “alternative” component becomes a critical barrier, because many XR systems still implicitly assume reliable motor access (e.g., hand controllers, precise gesture, or touch selection). Therefore, rather than broadly surveying XR-based AAC, the focus of this paper is refined to examine whether XR can be combined with motor-independent access technologies to enable alternative communication. In particular, this review focuses on whether XR-integrated Brain-Computer Interfaces (BCIs) based AAC devices exist for people who rely on AAC (e.g., CP, ASD, ALS and other speech disorders), and what advantages, usefulness and usability evidence is available.

BCI is a technology that enables direct communication between the human brain and external devices without relying on peripheral nerves (Bularka and Gontean, 2016). P300, which utilises the natural responses of people’s attention (Spencer and Polich, 1999), is a common instance of BCI signals (Wu et al., 2019). The fundamental mechanism of P300 is based on the feature of Electroencephalogram (EEG) where amplitude shift will happen after 300 milliseconds from an oddball visual or auditory stimulus (Başar et al., 1984; Yagi et al., 1999). Different from P300 which is a passive way of using brain signals (Mertens and Polich, 1997), Motor-Imagery (MI) is an active BCI interaction (Zander et al., 2010). MI-based BCI was also used in conventional AAC interfaces (Brumberg et al., 2016). Importantly, BCI has a direct connection to AAC because P300-based spelling interfaces have been a canonical communication paradigm since early “mental prosthesis” work (Farwell and Donchin, 1988), and BCI-AAC has been continuously discussed as an access pathway for people with severe disabilities (Wolpaw et al., 2000; Orlandi et al., 2021). The key question for this review is not whether BCI can work in general, but whether BCI can be operationalised within XR as a practical AAC access method for people who rely on AAC.

A few studies implied BCI uses in VR for cases with CP (Taherian et al., 2016, 2017), attention deficit hyperactivity disorder (ADHD) (Wang et al., 2007) and neuromuscular disorders as assist technologies (Osama and Aslam, 2020) by using Emotiv’s headset. Meanwhile, Kansaku (2015) enumerated their works of AR-based BCI for people with complex needs to regain control. Although the ultimate goal of Kansaku (2015) was not for communication, the transferable mechanism suggests that intent decoding and command output in AR/VR could be repurposed to serve alternative communication. In addition, Käthner et al. (2015) directly examined P300-based BCI typing in a VR setup, indicating that a classic AAC-style BCI interface can be deployed in an immersive display. Despite rapid growth in XR-based communication supports, most systems still assume reliable motor access (touch, gesture, controller use, or stable gaze), leaving users with severe motor impairment without a practical pathway to alternative communication. Meanwhile, BCIs provide motor-independent access for AAC in conventional displays, but it remains unclear whether and how BCI-mediated AAC has been operationalised within immersive XR environments, and what evidence exists for usability and usefulness in people who rely on AAC. Therefore, we systematically reviewed studies integrating XR (VR/AR/MR) with BCI to support AAC-relevant communication for people who rely on AAC, synthesising evidence on feasibility, usability, and usefulness outcomes.

## 2 Methods

### 2.1 Study design

All types of studies were included in this review, such as clinical trials, trials, case studies, single case studies, single-case experimental studies etc. Reviews, conference abstracts without full text, lecture slides, proof of concept and posters were excluded in this study.

### 2.2 Participants

The target participant group was people who rely on AAC or have at least one type of speech or communication disorder. However, the cause and severity of the speech or communication disorder were not limited. This study intended to include all people who rely on AAC. The related diseases, disorders, conditions or healthcare domains are cerebral palsy, autism spectrum disorder, amyotrophic lateral sclerosis, speech disorders, dystonic disorders, mutism, aphasia and articulation disorders.

Studies were required to include at least one target clinical participant group relevant to AAC use or communication disability. Typically developed participants were included only when they served as a comparator or control group within studies that also included target clinical participants. Findings from typically developed participants were therefore interpreted as comparator data rather than direct evidence of clinical applicability for AAC users.

### 2.3 Interventions

The intervention was limited to XR-integrated BCI based AAC systems. In this review, XR is used as an umbrella term for virtual reality (VR), augmented reality (AR), and mixed reality (MR), rather than referring to VR alone. The intervention must include information output from the participant. Communication training or rehabilitation interventions were excluded when they only aimed to improve communication-related skills, engagement, speech, or social behaviour, without providing an AAC output pathway through BCI. For example, interventions that only create avatars to help people be more engaged in training or rehabilitation were excluded. Additionally, studies which claimed to be XR based but were not in an immersive environment were excluded from this review.

### 2.4 Comparators

XR could be compared with conventional interfaces such as desktops or LED/LCD screen displays. However, the comparison was not a compulsory element in this study.

### 2.5 Systematic review protocol

The overall procedures of this systematic review included: defining search strategy and data source, retrieving papers from the data sets, removing duplicates, screening the titles and abstracts, reviewing the articles and proposing the inclusion and exclusion, assessing the eligibility and then extracting the data and analysing risks for included papers.

After retrieving papers from the data sets, Covidence was used as the review management system. The duplicates were firstly automatically detected by the Covidence system and then a manual duplicates removal was performed in the screening and review sections. Both screening and reviewing were done by two authors of this paper. If there were conflicts, another reviewer would be asked for a third view. Nevertheless, the final decisions for the conflicts were made after a discussion by all three reviewers.

### 2.6 Search strategy

The overall search strategy was a combination of Participants (P), Interventions (I), and Outcomes (O). As the comparison was not the focus of this study, there was no restriction keyword regarding Comparators (C). The search strategy included several neural disabilities/disorders and their related speech disorder syndromes as participants, VR and its synonyms as the first intervention, BCI and its related terms as the second intervention. And the outcome terms focused on communication, AAC, assistive communication, communication access, and related communicative output concepts. The full list of keywords and other details of search strategies were included in the Supplementary documents.

### 2.7 Data sources

The searches were conducted in MEDLINE (Ovid MEDLINE(R) ALL), Embase (Ovid interface), Web of Science, Scopus, and CINAHL Plus with Full Text (EBSCOhost interface). The initial search was completed on 30 June 2021, and the search was last updated on 18 November 2025. No publication-date restrictions were applied. The extensive list of keywords, subject headings and topics was selected based on the concept of speech impairment as the population, VR and its related terms as the first intervention, BCI and related technologies as the second intervention, and communication as the outcome. No language restriction was applied at the database search stage because language-filter functions vary across bibliographic databases and search engines. However, English was defined *a priori* as the target language for eligibility assessment to ensure consistency in screening, study selection, and data extraction. This language restriction should be considered when interpreting the comprehensiveness of the review.

### 2.8 Study selection

The inclusion criteria were: 1. The paper must involve people with cerebral palsy / people with autism / people with at least one type of speech impairments; 2. The device used in the study must be based on AR/VR/MR; 3. The study must involve at least one BCI-related technology; 4. The study had to provide an AAC-relevant output pathway, such as letter, word, symbol, sentence, message, or speech-output generation controlled through BCI; 5. The full-text article had to be available in English.

Studies were excluded if: 1. The population was people with a motor impairment only; 2. VR/AR/MR was only mentioned but not a core part of the design; 3. There was no BCI technology involved; 4. The outcome was completely movement/motor rehabilitation instead of communication alternation; 5. Studies were excluded if BCI was used only for neurofeedback, motor rehabilitation, speech therapy, symptom reduction, or social-communication training without AAC message selection or communication output; 6. The paper only described devices for communication training purposes; 7. All reviews, conference abstracts without full-text paper, abstract or poster collections, lecture slides, or advertisement posts, were excluded.

### 2.9 Data extraction

The full text of the studies was peer-reviewed after the peer abstract screening. Each paper was extracted and assessed by two people. The extraction sheet was shared online among group members. The extracted data were organised into predefined domains to support structured comparison across studies. The studies’ demographic information of the participants, such as sample size, age of the sample, sex and disorder/disability diagnosis, were extracted to assess that the population is aligned with the purpose of the study. Additionally, Manual Ability Classification System (MACS) (Eliasson et al., 2006), Communication Function Classification System (CFCS) (Hidecker et al., 2011), Gross Motor Function Classification System (GMFCS) (Palisano et al., 2008) and inclusion/exclusion criteria were searched to understand the physical requirements of using the devices. If the studies explicitly illuminated the physical requirements or level/type of support required to use the device, the relevant information would also be listed in the data extraction sheet. The full-text content of each study was arranged into columns of study design, aims/objectives, tasks, comparisons, outcome measures, results, authors’ conclusions and study limitations. To answer the usefulness and usability research questions, this information was pulled: whether the study is a usability study, the study’s technology readiness level, technology/algorithms used in the study, the accuracy and information transfer rate (ITR), the study’s intervention characteristics, intervention key elements and setting, intervention time points, cost, whether the proposed device is customizable. The ITR was firstly introduced by Wolpaw et al. (2000) to evaluate the efficiency of a BCI system. It was initially calculated as transferred bits divided by time consumed. However, the different papers might have a different formula to measure it (Yuan et al., 2013). The original results from papers were transcribed and transformation would only be applied if necessary. The Oxford 2011 Levels of Evidence (LOE) was adopted in this review to estimate the quality of the study.

### 2.10 Data analysis

A descriptive synthesis was conducted according to the data extraction of the papers. The types of XR-integrated BCI-based AAC were summarised in tables. The information about interventions’ target group, time points, effectiveness, costs and limitations were synthesised in this review. Because of the small number of included studies and heterogeneity in populations, systems, tasks, and outcome measures, meta-analysis was not appropriate. Instead, we conducted a structured narrative synthesis including tabulation of key metrics such as accuracy, ITR, usability outcomes, participant characteristics, and direction of findings where available. There were no extra study candidates found in the references of the included full-text articles.

The risk of bias was a list of 14 peer-assessed questions based on Lee et al. (2008). There were four marks to reflect whether the component had satisfied the quality standards. If the paper illuminated the corresponding part sufficiently, two points would be given. If the aspect of the study was included but not sufficiently described, only one point would be given to the study. If it lack a certain element, zero points would be given. However, if the question was not related to the study, the N/A, stand for” Not Applicable” would be given. This N/A would not be ignored in the statistical analysis. In the end, the final point was calculated by average the points the paper was given.

## 3 Results

### 3.1 Studies retrieved

**Figure 1:**
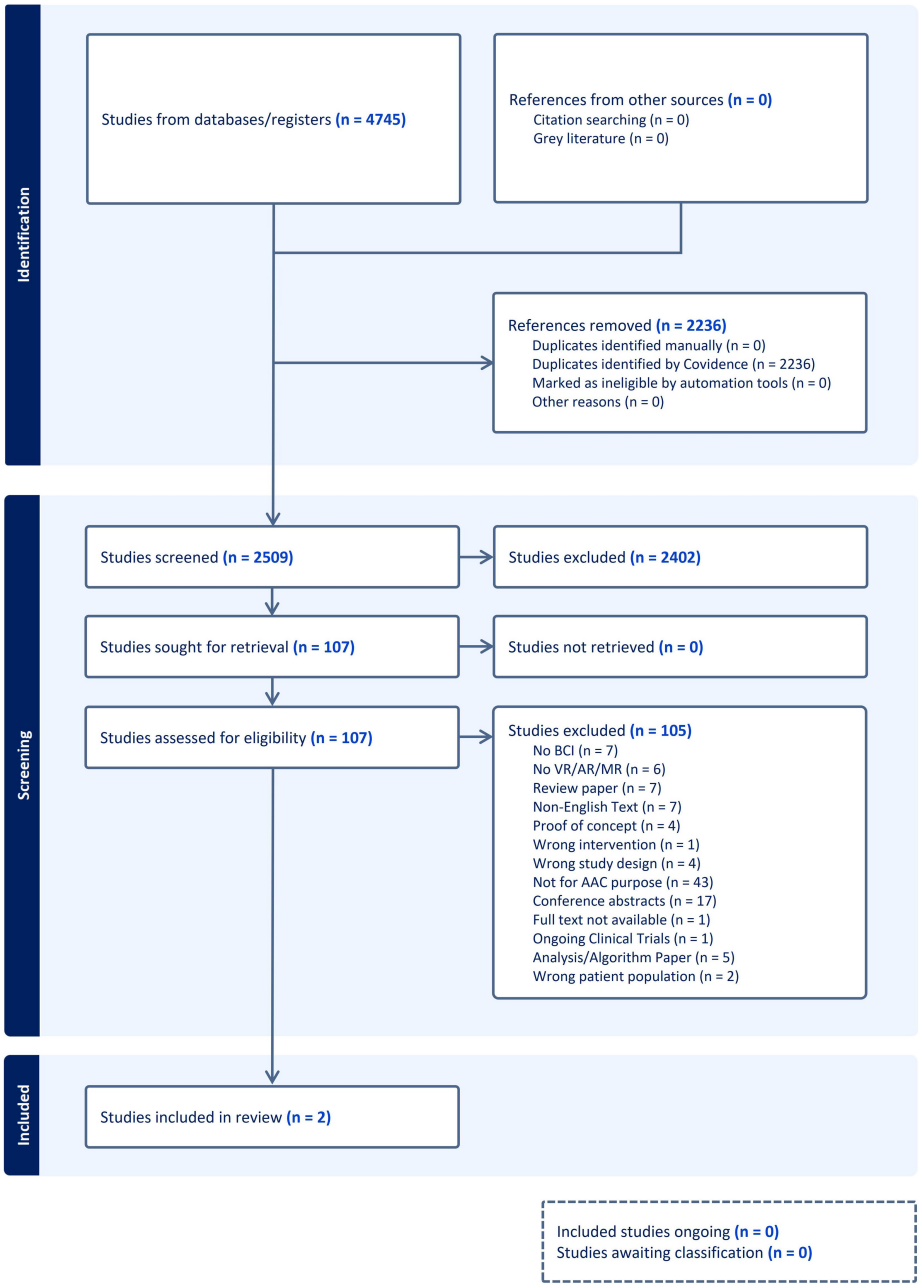
PRISMA flowchart of studies review results.

The studies were searched and retrieved by the 18th of November in 2025. There were 4,745 in total studies retrieved from databases listed above, and 2,236 duplicates were automatically detected and removed by the Covidence system. All authors in this study screened 2,509 papers’ titles and abstracts and filtered out 2,402 studies. The exclusion reasons were listed in Figure 1. The remaining 107 full-text articles were assessed for eligibility. Following full-text review and eligibility assessment, two studies met the final inclusion criteria and were included in the data extraction and synthesis.

### 3.2 Study selection and characteristics

Käthner et al. (2015) compared different setups for P300 based BCI keyboard typing. It laterally compared the interfaces between VR and conventional displays (Table 1). In addition, it also compared these setups between 18 typically developed participants and one person with ALS (Table 2), who was in Locked-in State (LIS). The purpose of this study was to investigate the usability of P300 BCI keyboard typing in a VR environment. The traditional P300 speller by Farwell and Donchin (1988) was adopted in this study. Both online classification, Stepwise Linear Discriminant Analysis (SWLDA), and offline analysis, Analysis of Variance (ANOVA), were employed in the study (Table 3). The results appeared to match accuracy and ITR. Lastly, the study Käthner et al. (2015) concluded that VR based P300 BCI keyboard typing has a comparable accuracy as the conventional displays. For the drawbacks of using VR, it raised the attention of isolation for long-time using of VR.

**Table 1:** Research articles of XR-integrated BCI based AAC for people who rely on it: content.

Study characteristic	Käthner et al. (2015)	Zhao et al. (2023)
Study design	Case study	multiple case study
Aims/objectives	To explore if similar P300-BCI based spelling performances can be achieved with a virtual reality (VR) headset compared to a conventional flat screen monitor.	To propose an MR-based wearable AAC (wAAC) and compare usability/acceptability between Eye-Gaze (EG) and BCI interaction options.
Tasks	Online spelling with a BCI using three different display methods	Compose sentences via the MR communication board; target templates included (a) I want [object] (b) Do you like [object] (c) I do not like [object]
XR modality	VR head-mounted display	MR head-mounted display
AAC task/output	P300-based letter spelling	Word selection, sentence generation, and speech output through an MR communication board
BCI paradigm	Visual P300 ERP speller	Visual P300-based selection from MR communication board
Comparisons	TD vs. ALS, Monitor vs. VR (Glass A, Glass B)	EG vs. BCI
Main outcome metrics	Online spelling accuracy, ITR, correctly selected letters per minute, ERP measures, spontaneous usability remarks	Accuracy, ITR/selections per minute, sensitivity, specificity for eye-gaze, QUEST 2.0, NASA-TLX, qualitative participant feedback
Results	TD: 94% (15.5 bits/min) using three flash sequences for spelling with the monitor and glasses A, 96% (16.2 bits/min) with glasses B. LIS: accuracy of 100% (10 bits/min) using the glasses A condition in one session. TD: one flash sequence is possible with the VR headset (mean:32.1 bits/min, maximum reached by one user: 71.89 bits/min at 100% accuracy).	EG: accuracy 93.30%; ITR 8.55 selections/min; sensitivity 98.78%, specificity 50%. BCI: online accuracy 5.91% (offline 9.28%); ITR ≈ 1.13 selections/min.
Conclusions	“VR headset allows for rapid P300 BCI communication in healthy users and may be a suitable display option for severely paralyzed persons.”	MR-based AAC was feasible for communication; EG showed high usability/acceptability, while the BCI option was “questionable” in the current implementation but may still be worth further investigation as an access method for users with severe motor limitations.

**Table 2:** Research articles of XR-integrated BCI based AAC for people who rely on it: demographic.

Participant characteristic	Käthner et al. (2015)	Zhao et al. (2023)
Sample size	19 (1 ALS and 18 TD)	10(2 CP and 8 TD)
Age of sample	TD: 21–34 yrs., mean = 25 ± 3.9 yrs., ALS: 80	TD: 24–44 yrs., CP: 23–32 yrs
Gender	TD: 10F/8M ALS: 1F	TD: 2F/6M CP: 1F/1M
Diagnosis	ALS with locked-in state	CP
Clinical functional classification	ALS Functional Rating Scale score = 0	MACS level II CFCS level I GMFCS level I
Prior AAC/BCI experience	ALS participant communicated using horizontal eye movement; prior BCI/AAC experience not fully reported	Prior AAC/BCI experience varied: one CP participant had no AAC experience and one had previous BCI-AAC research experience

**Table 3:** Research articles of XR-integrated BCI based AAC for people who rely on it: technical information.

Technical characteristic	Käthner et al. (2015)	Zhao et al. (2023)
Interaction method	P300 BCI	Eye-gaze, P300 BCI
Algorithms/interfaces	BCI2000, SWLDA, ANOVA	HoloLens 2, YOLOv5, LDA
XR hardware	Oculus Development Kit 1 VR headset	Microsoft HoloLens 2
EEG setup	8 active electrodes; g. USBamp; BCI2000	22 EEG channels using g. Nautilus SAHARA
Classification/algorithm	SWLDA for online classification	LDA for online BCI classification; offline EEGNet analysis
Communication output	Letter spelling	Word selection, sentence construction, and speech output
BCI accuracy	TD(Monitor, 3 flashes): 94%, TD(Glass A, 3 flashes): 94%, TD(Glass B, 3 flashes): 96%, TD(Not Specified, 1 flash): 64%, ALS(Monitor) N/A due to low accuracy, ALS (VR) 70%	EG: accuracy 93.30%, sensitivity 98.78%, specificity 50.00%. BCI: online accuracy 5.91%, offline accuracy 9.28% (chance 3.85–4.35%).
BCI speed/ITR	TD(Monitor, 3 flashes): 15.5 bits/min, TD(Glass A, 3 flashes): 15.5 bits/min, TD(Glass B, 3 flashes): 16.2 bits/min, TD(Not Specified, 1 flash): 32.1 bits/min, ALS: 10 bits/min in one successful VR session	Avg ITR: EG 8.55 selections/min (CP 6.52; TD 9.42); BCI 1.13 selections/min.
Non-BCI comparator	Conventional monitor	Eye-gaze access
Usability/acceptability	Spontaneous usability comments only; no standardized usability questionnaire reported	QUEST 2.0 and NASA-TLX reported. Eye-gaze showed favorable usability and workload; BCI usability was questionable
Main technical limitation	VR headset comfort and display quality concerns	Low BCI accuracy; time synchronization, signal quality, and online classification limitations

Building on this foundational VR P300-speller setup, Zhao et al. (2023) shifted the focus toward a wearable mixed-reality AAC system and directly contrasted eye-gaze with BCI as access methods. Zhao et al. (2023) developed a wearable mixed-reality AAC (wAAC) system that integrates computer vision with two interaction modalities—eye-gaze (EG) and a P300-based BCI—aiming to support communication by enabling users to select recognized objects in the environment through an MR headset. The study used a multiple case study design and recruited eight neurotypical participants and two participants with CP to evaluate usability, acceptability, and performance across the two access methods. In the EG condition, the system achieved a mean selection accuracy of 93.30% and an average speed of 8.55 selections/min; user-reported usability and workload were favorable (QUEST 2.0 mean 4.30; NASA-TLX mean 2.14). By contrast, the BCI condition showed substantially lower mean accuracy (reported as 9.28%), leading the authors to characterize the BCI usability as questionable in its current form, while highlighting the promise of the EG-based MR AAC approach.

### 3.3 Synthesized findings

#### 3.3.1 Technical characteristics

Both included studies used EEG-based visual P300 BCI paradigms, but the systems differed substantially in display modality, interaction design, AAC output, and level of ecological integration. Käthner et al. (2015) used BCI2000 and SWLDA for online P300 spelling through a VR head-mounted display. Zhao et al. (2023) used Microsoft HoloLens 2, YOLOv5 object detection, a g. Nautilus EEG system, and linear discriminant analysis (LDA) for P300-based BCI selection within a wearable MR-AAC system.

#### 3.3.2 Structured summary of findings

Because only two studies met the final inclusion criteria, findings were synthesized using structured tables and descriptive directional comparisons across predefined outcome domains. Table 4 summarises the included studies across evidence base, AAC output, BCI paradigm, XR modality, BCI performance, speed/ITR, usability and acceptability, participant characterization, and clinical relevance.

**Table 4:** Research articles of XR-integrated BCI based AAC for people who rely on it: structured summary of findings.

Review domain	Summary of findings
Evidence base	Two studies were included, with 29 participants in total. 26 of 29 participants were healthy or neurotypical, 3 participants represented target clinical populations: 1 ALS-LIS participant and 2 CP participants.
AAC output	Both studies provided direct communication output. Käthner et al. (2015) used letter spelling, whereas Zhao et al. (2023) used word selection, sentence construction, and speech output.
BCI paradigm	Both studies used visual P300-based EEG BCI.
XR modality	Käthner et al. (2015) used VR as a head-mounted display for P300 spelling. Zhao et al. (2023) used MR to present an AAC communication board integrated with object detection.
BCI performance	Käthner et al. (2015) reported high spelling accuracy in controlled VR P300-speller conditions, while Zhao et al. (2023) reported low online BCI accuracy in a wearable MR-AAC prototype.
Speed/ITR	Both studies reported speed-related metrics, but units were not directly comparable. Käthner et al. (2015) reported ITR in bits/min, while Zhao et al. (2023) reported selections/min.
Usability and acceptability	Usability reporting was inconsistent. Zhao et al. (2023) reported standardized measures including QUEST 2.0 and NASA-TLX, whereas Käthner et al. (2015) reported only spontaneous participant comments on usability.
Participant characterization	Participant characteristics were reported using disease-specific descriptors. Zhao et al. (2023) reported MACS, CFCS, and GMFCS for the CP participants. Käthner et al. (2015) reported ALS-LIS status and ALS Functional Rating Scale score.
Clinical relevance	Clinical representation was limited. Käthner et al. (2015) included one severely paralyzed ALS-LIS participant, while Zhao et al. (2023) included two participants with CP who were GMFCS I, CFCS I, and verbally communicative. Therefore, evidence for people who rely heavily on AAC remains preliminary.

#### 3.3.3 Performance outcomes

The two included studies showed markedly different levels of BCI performance. Käthner et al. (2015) demonstrated that P300 spelling through a VR headset could achieve performance comparable to a conventional monitor in controlled group: with three flash sequences, accuracy was 94% in both the monitor and whole-matrix VR condition, and 96% when individual letters filled the VR field of view. The corresponding ITR values were 15.5 bits/min for the monitor and whole-matrix VR conditions and 16.2 bits/min for the single-letter VR condition. In an additional one-flash VR spelling task, healthy participants achieved a mean accuracy of 64% and an ITR of 32.1 bits/min. In the ALS-LIS participant, one VR headset session reached 100% online spelling accuracy with an ITR of 10 bits/min, although the single-case nature of this evidence limits generalizability.

In contrast, Zhao et al. (2023) reported substantially weaker performance for the BCI pathway in a wearable MR-AAC prototype. The eye-gaze condition achieved an average accuracy of 93.30% and an average speed of 8.55 selections/min. However, the BCI condition achieved only 5.91% online accuracy and 9.28% offline accuracy, with an average speed of 1.13 selections/min. Because the reported BCI chance level varied from 3.85 to 4.35%, the online BCI performance was only slightly above chance and was unlikely to support practical AAC use in its current implementation. This contrast indicates that P300-BCI communication may perform well in controlled spelling paradigms, but translation to wearable MR-based AAC remains technically immature.

#### 3.3.4 Usability, acceptability, and practical constraints

Käthner et al. (2015) recorded spontaneous comments from five healthy participants. Some comments supported the VR display, including one participant who found it easier to maintain visual focus using the VR headset than the monitor. However, four participants reported negative aspects of the VR headset, including pixelated display quality, out-of-focus letters at the matrix margins, possible heaviness during prolonged use, and warmth under the headset.

Zhao et al. (2023) provided more structured usability and acceptability data. The eye-gaze condition showed favorable usability and workload outcomes, including high QUEST 2.0 scores and low NASA-TLX workload scores. However, the BCI pathway showed substantially lower accuracy and slower selection speed, suggesting that the main barrier was not the MR-AAC concept but the maturity of the BCI access method. These findings highlight the need for rapid optimization of BCI signal acquisition, classification, calibration, and integration within XR-based AAC systems.

### 3.4 Quality assessment and risk of Bias

Based on the reviewers’ evaluation (Table 5), Käthner et al. (2015) and Zhao et al. (2023) were usability studies. Both studies were rated as low-level evidence, with Oxford 2011 LOE (Avg. Rated) = 4 for all papers (Table 5), and the Technology Readiness Level (1–9) was rated around prototype validation (Käthner: 5/5; Zhao: 5/5; Table 5).

**Table 5:** Research articles of XR-integrated BCI based AAC for people who rely on it: reviewer’s evaluation.

References reviewer^1^	Käthner et al. (2015)		Zhao et al. (2023)	
	R1	R2	R1	R2
Oxford LOE (Avg. rated)	4	4	4	4
Technology readiness level (1–9, Avg. rated)	5	5	5	5
Usability study	Yes	Yes	Yes	Yes

^1^The papers were randomly assigned. The reviewer number is just an indicator of different reviewers. The specific number does not refer to a specific reviewer.

The reviewer’s average quality scores were 78.86 for Käthner et al. (2015), 88.64 for Zhao et al. (2023), (Table 5). The quality grades were Good for Käthner et al. (2015) and Strong for Zhao et al. (2023) (Table 6). Overall, the inter-reviewer exact agreement across the checklist was 85.71% (Table 6).

Across the two studies, the questions/objectives and overall study designs were consistently described with sufficient clarity, and outcome measures were generally well defined with assessment procedures reported (Table 6). In contrast, common limitations were observed in the relatively small sample sizes, incomplete reporting of variance/precision for key outcomes, and limited explicit consideration of potential confounding factors (Table 6). Käthner et al. (2015) received comparatively lower ratings in the description and appropriateness of participant selection and in the reporting of participant characteristics, which is consistent with the study’s more limited representation of the target clinical population relative to the other included studies (Table 6).

**Table 6:** Assessment of risk of bias.

References reviewer^1^	Käthner et al. (2015)		Zhao et al. (2023)	
	R1	R2	R1	R2
Question/objective sufficiently described?	2	2	2	2
Study design evident and appropriate?	2	2	2	2
Method of subject/comparison group selection or source of information/input variables described and appropriate?	1	1	2	2
Subject (and comparison group, if applicable) characteristics sufficiently described?	1	1	2	2
If interventional and random allocation was possible, was it described?	N/A	N/A	N/A	N/A
If interventional and blinding of investigators was possible, was it reported?	N/A	N/A	N/A	N/A
If interventional and blinding of subjects was possible, was it reported?	N/A	N/A	N/A	N/A
Outcome and (if applicable) exposure measure(s) well defined and robust to measurement/misclassification bias? Means of assessment reported?	2	2	2	2
Sample size appropriate?	1	1	1	1
Analytic methods described/justified and appropriate?	2	2	2	2
Some estimate of variance is reported for the main results?	2	1	1	1
Controlled for confounding? (did they consider other factors playing a role – that may have a role on the results)	N/A	1	1	2
Results reported in sufficient detail?	2	2	2	2
Conclusions supported by the results?	2	1	2	2
Score (%)	85	72.73	86.36	90.91
Avg. score (%)	78.86		88.64	
Quality grade	Good		Strong	

^1^The papers were randomly assigned. The reviewer number is just an indicator of different reviewers. The specific number does not refer to a specific reviewer.

^2^Criteria were scored either 2 or 1 or 0 (2 = yes, 1 = partial, 0 = no) or if the criteria were not applicable to the paper it was scored N/A (not applicable). To make them comparative, overall scores are presented as a %. Quality grade: limited (score of ≤50%), adequate (>50% and ≤70%), good (>70% and ≤80%), strong (>80%) (Lee et al., 2008).

With respect to random allocation and blinding procedures, randomisation was not applicable across the included evidence base (Table 6). Blinding was treated as not applicable in Zhao et al. (2023).

Finally, results were reported in sufficient detail in both studies, and the conclusions were generally supported by the reported findings (Table 6). One reviewer rated the conclusions of Käthner et al. (2015) more conservatively, which may reflect the constraints associated with the limited target-population evidence available within that study (Table 6).

## 4 Discussion

### 4.1 Summary of main findings

The present review elucidated the feasibility, usability, and potential usefulness of XR-integrated BCI approaches for supporting communication-related functions in people who rely on AAC, while also highlighting marked heterogeneity in application targets and evaluation endpoints. The two included studies represented distinct integration pathways across immersive environments.

Käthner et al. (2015) examined an immersive-display implementation of a classic P300 speller and indicated that VR presentation can achieve communication performance comparable to conventional displays, with feasibility further demonstrated in a participant with ALS. Zhao et al. (2023) moved from a laboratory speller toward a wearable mixed-reality AAC concept and directly contrasted eye-gaze and a P300-based BCI as access methods. The findings suggested that the MR-AAC concept was feasible and acceptable, but that the BCI access pathway, at least in the reported implementation—remained a major bottleneck compared with eye-gaze in terms of practical communication performance.

Taken together, the evidence base remains narrow and largely exploratory, yet it supports the notion that immersive XR environments can host brain-driven interaction paradigms for AAC-related purposes. The main contribution of the current review is therefore not to establish clinical effectiveness, but to clarify the current state of an emerging field. The findings indicate cautious evidence of technical feasibility, while also identifying a translational gap between controlled BCI in immersive displays and practical AAC use in real-world XR environments.

### 4.2 BCI performance and AAC feasibility

The contrast between the two included studies is central to interpreting the field. Käthner et al. (2015) demonstrated that P300 spelling can be implemented successfully through a VR headset. Healthy participants achieved high online spelling accuracy across VR display conditions, and one ALS-LIS participant achieved successful online spelling in a VR headset session. This supports the view that immersive or head-mounted displays can provide a viable stimulus-presentation layer for BCI-mediated communication.

In Zhao et al. (2023), however, BCI performance was lower when implemented in a wearable MR-AAC prototype. The eye-gaze condition achieved high accuracy and favorable usability, whereas the BCI condition achieved low online accuracy and slow selection speed. The contrast between the successful eye-gaze pathway and the limited BCI pathway indicates that the main challenge lies in the current maturity of BCI access within XR-based AAC systems, rather than in the XR-AAC concept itself.

This distinction is important for future development. XR may increase privacy, portability, contextual vocabulary access, and ecological relevance. However, these benefits can only be fully realized if BCI access becomes sufficiently reliable, efficient, and comfortable under real-world use conditions. Therefore, XR-BCI-AAC research should therefore prioritize improvements in signal acquisition, classification accuracy, calibration efficiency, cognitive workload, fatigue management, headset comfort, and hardware integration.

### 4.3 Standardization of outcome metrics

The included studies reported partially overlapping but non-equivalent outcome metrics, which limited direct comparison and prevented quantitative pooling. Although both studies reported accuracy, the Käthner et al. (2015) reported spelling accuracy in a controlled P300 letter-selection paradigm with ITR in bits/min, whereas Zhao et al. (2023) reported selection accuracy within a wearable MR-AAC system in selections/min. Because these measures reflect related but different constructs, they were compared descriptively rather than pooled quantitatively.

The findings of this review suggest that outcome measurement in XR-BCI-AAC is still underdeveloped. Both Käthner et al. (2015) and Zhao et al. (2023) metrics alone are insufficient to capture practical communication value. Therefore, the issue is not only that existing studies used different metrics, but that current measurement approaches are not yet fully suited to the developmental status and functional complexity of XR-BCI-AAC systems. Future XR-BCI-AAC studies should develop stage-appropriate evaluation frameworks that distinguish early technical feasibility, prototype usability, and real-world AAC communication, while assessing both BCI reliability and meaningful communication outcomes.

### 4.4 Clinical relevance and participant characterization

The two included studies provided clinically relevant but disease-specific participant descriptions. Käthner et al. (2015) included one participant with ALS in locked-in state, reported an ALS Functional Rating Scale score of 0, and described that the participant communicated using horizontal eye movements. These details indicate severe motor impairment and support the clinical relevance of this single case for motor-independent communication access. In contrast, Zhao et al. (2023) reported CP-specific functional classifications for the two participants with CP, including GMFCS I, CFCS I, suggesting relatively mild functional profiles and preserved verbal communication.

The ALS participant was described using ALS-LIS status and ALS Functional Rating Scale score, whereas the CP participants were described using CP-specific functional classification systems. This disease-specific reporting is clinically appropriate, but it also shows why future XR-BCI-AAC research should move beyond diagnosis-specific feasibility and examine cross-condition transferability. Rather than treating participant heterogeneity only as a limitation, it should be considered a central design requirement: XR-BCI-AAC systems should be adaptable to different motor, sensory, cognitive, and communication profiles. Therefore, the goal is not simply to build a device for one diagnostic group, but to develop flexible communication technologies that reduce physical access barriers and support shared communication across heterogeneous users.

### 4.5 Relevant studies

Although these studies did not meet the final AAC-output criterion, they expand the future horizon of XR-BCI-AAC by showing how immersive environments, neurofeedback, wearable BCI, and real-time command output may be redirected toward more inclusive communication technologies Table 7.

**Table 7:** Related studies and their relevance to XR-integrated BCI-AAC.

Study	Technology profile	Primary purpose	Reason for exclusion from primary synthesis	Relevance to discussion
Ehrlich et al. (2025)	VR + closed-loop EEG-based BCI neurofeedback	Rehabilitation of isolated focal laryngeal dystonia voice symptoms	Did not provide AAC message selection, spelling, word/sentence output, or AAC interface control	Demonstrates how immersive VR and BCI neurofeedback can be used in communication-related clinical rehabilitation, but represents symptom-oriented therapy rather than AAC access
Arpaia et al. (2022)	Wearable AR + SSVEP-based BCI + robot control	ADHD rehabilitation through robot-assisted therapy	BCI output controlled robot movement rather than communicative AAC output	Shows that wearable AR can deliver BCI stimuli and real-time command output in a portable setup, which may inform future XR-BCI-AAC interaction design
Contrada et al. (2024)	Computer-based VRRS language rehabilitation + tDCS	Aphasia rehabilitation after stroke	Did not use BCI and did not provide AAC access or message output	Illustrates the broader role of XR/computer-based environments in intensive language and communication rehabilitation
Mandapati and Ranjan (2025)	VR-based audio-visual brainwave entrainment + EEG measurement	Attention and learning intervention in children with ADHD	Did not use BCI control and did not target AAC communication output	Provides distant adjacent evidence that VR can deliver controlled neurostimulation and EEG-based evaluation, but is not AAC evidence

Ehrlich et al. (2025) is particularly relevant because it combines VR, EEG-based BCI, and a communication-related disorder. The study used realistic VR speaking scenarios and closed-loop neurofeedback to help patients with isolated focal laryngeal dystonia modulate abnormal brain activity during speech. Although this is not AAC evidence, it demonstrates how XR can provide ecologically meaningful contexts for BCI-mediated intervention.

Arpaia et al. also provides useful design insight. Their wearable AR-based SSVEP-BCI system enabled children undergoing ADHD therapy to control a social robot through flickering icons displayed on AR smart glasses. The system demonstrates a portable AR-BCI command-output pipeline, but the output was robot control for rehabilitation rather than expressive AAC communication. From an AAC perspective, this type of system may provide design inspiration for portable AR-based BCI interaction.

Two additional studies used XR together with non-BCI neuromodulation/entrainment paradigms rather than classical BCI decoding, and can be considered as a parallel line of “XR + neurostimulation” approaches. Contrada et al. and Mandapati and Ranjan show how XR or computer-based environments can support neurostimulation, or EEG-based evaluation.

These adjacent studies suggest design possibilities for future XR neurotechnology, while confirming the need to distinguish AAC access from broader communication-related rehabilitation. They show that XR can provide realistic interaction contexts, wearable systems can support portable neurotechnology, and EEG/BCI-based feedback pipelines can be redirected toward communication-oriented applications.

### 4.6 Future suggestions

Based on the review results, gaps in device design, study design, and innovation directions were identified. Nevertheless, XR-BCI-AAC offers distinctive potential as immersive, intuitive and engaging communication approach that may support more context-rich interaction than conventional interfaces. As a result, future XR-BCI-AAC research should move from proof-of-concept feasibility toward clinically meaningful communication outcomes.

From the device-design aspect, the present evidence suggests that access-method selection remains decisive. In Zhao et al. (2023), eye-gaze clearly outperformed the reported BCI implementation, implying that near-term wearable XR-AAC may rely more on robust non-BCI access, while BCI remains a longer-term pathway. Conversely, the AR-SSVEP demonstrates that portable BCI pipelines can support wearable XR interaction with lower training burden and real-time feedback. Future XR-AAC systems may therefore benefit from hybrid strategies that combine reliable access methods, such as gaze, with BCI as a complementary channel until BCI interaction reaches a higher level of accuracy and reliability. Accordingly, further investigation of neural mechanisms, signal features, and decoding algorithms is essential to improve the accuracy, robustness, and responsiveness of BCI-based interaction in XR-AAC contexts.

From the study-design perspective, the current evidence base remains limited, with small sample sizes, methodological heterogeneity, and largely low-level study designs. Future research should include terminal users as participants, such as people with severe CP, ALS, locked-in syndrome, brainstem stroke, or other conditions involving severe motor or speech impairment. Although studies with healthy participants remain useful for technical development, they are insufficient for evaluating clinical AAC applicability. Stronger validation will require larger-scale studies with clearer participant stratification and harmonized reporting frameworks. Beyond accuracy and speed, future studies should report setup time, calibration burden, headset comfort, fatigue, error correction, user satisfaction, communication partner feedback, and long-term use. For AAC-specific systems, the key question should be not only whether users can select a target, but whether the system enables efficient, meaningful, and acceptable communication.

For innovation, future XR-based AAC should be originated from the target group’s perspective, emphasising not only feasibility but also long-term acceptability and participation impact. XR affords new opportunities for contextualised and motivating interaction. However, if the interface is not adaptive to sensory-motor-cognitive constraints, the novelty of XR may increase rather than reduce barriers. Therefore, next-generation systems would be recommended to be not only adaptive but also educational and heuristic, aligning communication training with meaningful real-life contexts while maintaining low burden and high robustness.

### 4.7 Limitations

This review has limitations. Only two studies met the final eligibility criteria, and these studies differed in may perspectives. As a result, meta-analysis was not appropriate, and the synthesis was necessarily structured and descriptive.

The included studies also had limited clinical representation. Most participants were healthy or neurotypical, and only three clinical participants were included across the evidence base. Functional classifications and AAC-related participant descriptors were not consistently reported, which limited assessment of applicability to people who rely heavily on AAC.

Outcome heterogeneity was another limitation. Although both studies reported accuracy and speed-related outcomes, these metrics were not directly comparable across studies. Standardized usability measures were reported in only one study. In addition, the English-language eligibility criterion may also have affected comprehensiveness.

These limitations should be interpreted in the context of an emerging research area. The small evidence confirms the need for novelty of XR-BCI-AAC and provides a timely foundation for guiding future research toward clearer study boundaries, stronger reporting standards, and clinically meaningful implementation.

## 5 Conclusion

This review shows that XR-integrated BCI-based AAC is an emerging with substantial potential to expand communication access. The available evidence suggests that XR environments can support BCI-mediated communication interfaces, particularly in controlled P300 spelling paradigms. However, the gap between current XR-AAC prototypes and the promise of BCI-enabled XR-AAC highlights a growing need for research that advances BCI reliability, integration, and usability in context-rich communication environments.

The review contributes by identifying the current translational gap and proposing priorities for future evaluation. Future work should focus on clinically representative AAC users, standardized outcome metrics, hybrid access methods, and long-term usability in real communication contexts. At present, XR-integrated BCI-AAC should be considered a promising early-stage direction rather than an established clinical solution.

## Data Availability

The original contributions presented in the study are included in the article/Supplementary material, further inquiries can be directed to the corresponding authors.
