# Supplementary material for: XR-integrated brain-computer interfaces for augmentative and alternative communication: a systematic review
Source: Front Hum Neurosci. 2026 Jul 15;20:1842938. doi: 10.3389/fnhum.2026.1842938 (PMC13416351; doi:10.3389/fnhum.2026.1842938)
Supplement: Supplementary file 1 [file Data_Sheet_1.PDF]

Supplementary Material

1 Supplementary Data

1.1 Search Strategy

Table S1. Search Strategy - Scopus

Participants (P)	Intervention-1 (I-1)	Intervention-2 (I-2)	Outcomes (O)
TITLE-ABS-KEY ("cerebral palsy" OR "amyotrophic lateral sclerosis" OR "speech disorder*" OR "dystonic disorder*" OR "mutism" OR "akinetic mutism" OR "aphasia" OR "speech sound disorder*" OR "communication disorder*" OR cp OR als OR "autism" OR "autism spectrum disorder" OR "asperger syndrome" OR "autistic disorder" OR asd)	TITLE-ABS-KEY ("augmentative and alternative communication" OR "assistive technology" OR "communication aid*" OR "control* technolog*" OR "brain computer interface*" OR "brain machine interface*" OR "electroencephalograph*" OR "electrocorticograph*" OR "electromyograph*" OR "surface electromyograph*" OR "electrodermal response" OR "electrodermal" OR "galvanic skin response" OR "eye track*" OR "gaze" OR "eye fixation" OR "eye gaz*" OR "infrared sens*" OR "electrooculograph*" OR "oculograph*" OR "head orient*" OR "voice recogni*" OR "hearable*" OR "mechanical switch" OR "electromagn*" OR aac OR bci OR eeg OR ecog OR emg OR semg OR egt)	TITLE-ABS-KEY ("virtual reality" OR "augmented reality" OR "mixed reality" OR vr OR ar OR mr)	TITLE-ABS-KEY ("communicat*" OR "language ability" OR "speech rehabilitation" OR "speech" OR "speech and language rehabilitation" OR "facilitated communication" OR "social")

[a] TITLE-ABS-KEY: Search keys in title, key words and abstract.

Table S2. Search Strategy - CINAHL

Participants (P)	Intervention-1 (I-1)	Intervention-2 (I-2)	Outcomes (O)
TX(cerebral palsy) OR<br>TX(amyotrophic lateral sclerosis) OR<br>TX(speech disorder*) OR<br>TX(dystonic disorder*) OR<br>TX(mutism) OR<br>TX(akinetic mutism) OR<br>TX(aphasia) OR<br>TX(speech sound disorder*) OR<br>TX(communication disorder*) OR<br>TX(autism) OR<br>TX(autism spectrum disorder*) OR<br>TX(asperger syndrome) OR<br>TX(autistic disorder*) OR<br>AB asd OR<br>AB cp OR<br>AB als	TX(augmentative and alternative communication) OR<br>TX(assistive technology) OR<br>TX(communication aid*) OR<br>TX(control* technolog*) OR<br>TX(brain computer interface*) OR<br>TX(brain machine interface*) OR<br>TX(electroencephalograph*) OR<br>TX(electrocorticograph*) OR<br>TX(electromyograph*) OR<br>TX(surface electromyograph*) OR<br>TX(electrodermal response) OR<br>TX(electrodermal) OR<br>TX(galvanic skin response) OR<br>TX(eye track*) OR<br>TX(gaze) OR<br>TX(eye fixation) OR<br>TX(eye gaz*) OR<br>TX(infrared sens*) OR<br>TX(electrooculograph*) OR<br>TX(oculograph*) OR<br>TX(head orient*) OR<br>TX(voice recogni*) OR<br>TX(hearable*) OR<br>TX(mechanical switch) OR<br>TX(electromagn*) OR<br>AB aac OR<br>AB bci OR<br>AB eeg OR<br>AB ecog OR<br>AB emg OR<br>AB semg OR<br>AB egt	TX(virtual reality) OR<br>TX(augmented reality) OR<br>TX(mixed reality) OR<br>AB vr OR<br>AB ar OR<br>AB mr	TX(communicat*) OR<br>TX(language ability) OR<br>TX(speech rehabilitation) OR<br>TX(speech) OR<br>TX(speech and language rehabilitation) OR<br>TX(facilitated communication) OR<br>TX (social)

[a] TX: Search keys in full text.

[b] AB: Search keys in abstract.

Table S3. Search Strategy - Web of Science

Participants (P)	Intervention-1 (I-1)	Intervention-2 (I-2)	Outcomes (O)
ALL=((cerebral palsy) OR (amyotrophic lateral sclerosis) OR (speech disorder*) OR (dystonic disorder*) OR (mutism) OR (akinetic mutism) OR (aphasia) OR (speech sound disorder*) OR (communication disorder*) OR (autism) OR (autism spectrum disorder*) OR (asperger syndrome) OR (autistic disorder*))<br>OR<br>AB=(cp OR als OR asd)	ALL=((augmentative and alternative communication) OR (assistive technology) OR (communication aid*) OR (control* technolog*) OR (brain computer interface*) OR (brain machine interface*) OR (electroencephalograph*) OR (electrocorticograph*) OR (electromyograph*) OR (surface electromyograph*) OR (electrodermal response) OR (electrodermal) OR (galvanic skin response) OR (eye track*) OR (gaze) OR (eye fixation) OR (eye gaz*) OR (infrared sens*) OR (electrooculograph*) OR (oculograph*) OR (head orient*) OR (voice recogni*) OR (hearable*) OR (mechanical switch) OR (electromagn*))<br>OR<br>AB=(aac OR bci OR eeg OR ecog OR emg OR semg OR egt)	ALL=((virtual reality) OR (augmented reality) OR (mixed reality))<br>OR<br>AB=(vr OR ar OR mr)	ALL=((communicat*) OR (language ability) OR (speech rehabilitation) OR (speech) OR (speech and language rehabilitation) OR (facilitated communication) OR (social))

[a] ALL: Search keys in Topic (Title, Abstract, Keywords, Keywords Plus), Author, Editor, Corporate Author, Group Author, Author, Identifier, Publication Name, DOI, Publication Year, Address, Organization-Enhanced, Conference, Language, Document Type, ISSN, Funding Agency, Funding Text, Grant Number, Accession Number, PubMedID

[b] AB: Search keys in only Abstract

Table S4. Search Strategy - Embase

Participants (P)	Intervention-1 (I-1)	Intervention-2 (I-2)	Outcomes (O)
cerebral palsy/<br>cerebral palsy.mp.<br>cp.tw.<br>autism/<br>autism spectrum disorder*.mp.<br>autism.mp.<br>asd.tw.<br>asperger syndrome/<br>asperger syndrome.mp.<br>autistic disorder/<br>autistic disorder.mp.<br>amyotrophic lateral sclerosis/<br>amyotrophic lateral sclerosis.mp.<br>als.tw.<br>speech disorder/<br>speech disorders.mp.<br>dystonic disorders/<br>dystonic disorders.mp.<br>mutism/<br>akinetic mutism/<br>mutism*.mp.<br>exp aphasia/<br>aphasia.mp.<br>speech sound disorder/<br>speech sound disorder.mp.<br>communication disorder/<br>communication disorder*.mp	(augmentative and alternative communication).mp.<br>aac.tw.<br>assistive technology/<br>assistive technolog*.mp<br>exp communication aid/<br>communication aid*.mp.<br>control* technolog*.mp<br>brain-computer interface/<br>brain-computer interface*.mp.<br>bci.tw.<br>brain-machine Interface*.mp.<br>electroencephalography/<br>electroencephalograph*.mp.<br>eeg.tw.<br>electrocorticography/<br>electrocorticograph*.mp.<br>ecog.tw.<br>electromyography/<br>electromyograph*.mp.<br>emg.tw.<br>surface electromyograph*.mp.<br>semg.tw.<br>electrodermal response/<br>electrodermal response.mp.<br>electrodermal.mp.<br>galvanic skin response.mp.<br>eye tracking/<br>eye track*.mp.<br>gaze/<br>eye fixation/<br>eye gaz*.mp.<br>egt.tw.<br>infrared sensor/<br>infrared sens*.mp.<br>electrooculography/<br>electrooculograph*.mp.<br>oculograph*.mp.<br>head orient*.mp.<br>voice recogni*.mp.<br>hearable*.mp.<br>mechanical switch.mp.<br>electromagn*.mp.	virtual reality/<br>virtual reality.mp.<br>vr.tw.<br>augmented reality/<br>augmented reality.mp.<br>ar.tw.<br>mixed reality.mp.<br>mr.tw.	interpersonal communication/<br>nonverbal communication/<br>language ability/<br>communicat*.mp.<br>speech rehabilitation/<br>speech/<br>"speech and language rehabilitation"/<br>facilitated communication/<br>speech.mp.<br>social.mp.

[a] mp.: Search for references where your words appear in several specific fields, including the title, abstract, subject heading, author keywords, and more.

[b] tw.: Search for references where your words appear in the title or the abstract only

Table S5. Search Strategy - Medline

Participants (P)	Intervention-1 (I-1)	Intervention-2 (I-2)	Outcomes (O)
cerebral palsy/	(augmentative and alternative communication).mp.	virtual reality/	communication/
cerebral palsy.mp.	aac.tw.	virtual reality.mp.	nonverbal communication/
cp.tw.	communication aids for disabled/	vr.tw.	communicat*.mp.
autism spectrum disorder/	communication aids for disabled.mp.	augmented reality/	social.mp.
autism spectrum disorder*.mp.	communication aid*.tw.	augmented reality.mp.	Communication Methods, Total/
autism.mp.	assistive technolog*.mp	ar.tw.	speech/
asd.tw.	control* technolog*.mp	mixed reality.mp.	speech.mp.
asperger syndrome/	brain-computer interfaces/	mr.tw.	
asperger syndrome.mp.	brain-computer interface*.mp.		
autistic disorder/	bci.tw.		
autistic disorder.mp.	brain-machine Interface*.mp.		
amyotrophic lateral sclerosis/	electroencephalography/		
amyotrophic lateral sclerosis.mp.	electroencephalograph*.mp.		
als.tw.	eeg.tw.		
speech disorders/	electrocorticography/		
speech disorder*.mp.	electrocorticograph*.mp.		
dystonic disorders/	ecog.tw.		
dystonic disorder*.mp.	electromyography/		
mutism/	electromyograph*.mp.		
mutism.mp.	emg.tw.		
exp aphasia/	surface electromyograph*.mp.		
aphasia.mp.	semg.tw.		
exp articulation disorders/	galvanic skin response/		
articulation disorder*.mp.	galvanic skin response.mp.		
	electrodermal response.mp.		
	electrodermal.mp.		
	fixation, ocular/		
	eye track*.mp.		
	eye gaz*.mp.		
	egt.tw.		
	infrared sens*.mp.		
	oculograph*.mp.		
	head orient*.mp.		
	Voice Recognition/		
	voice recogni*.mp.		
	hearable*.mp.		
	mechanical switch.mp.		
	electromagn*.mp.		

[a] mp.: Search for references where your words appear in several specific fields, including the title, abstract, subject heading, author keywords, and more.

[b] tw.: Search for references where your words appear in the title or the abstract only
